# A family-based intervention targeting parents of preschool children with overweight and obesity: conceptual framework and study design of LOOPS- Lund overweight and obesity preschool study

**DOI:** 10.1186/1471-2458-12-879

**Published:** 2012-10-17

**Authors:** Jenny Önnerfält, Lena-Karin Erlandsson, Kristina Orban, Malin Broberg, Christina Helgason, Kristina Thorngren-Jerneck

**Affiliations:** 1 Department of Paediatrics, Clinical Sciences Lund, Lund University, Lund, Sweden; 2 Vårdalinstitutet, Swedish Institute for Health Sciences, Lund University, Lund, Sweden; 3 Department of Health Sciences, Faculty of medicine, Lund University, Lund, Sweden; 4 Department of Psychology, University of Gothenburg, Gothenburg, Sweden; 5 Department of Psycholog, Lund University, Lund, Sweden; 6 Department of Paediatrics; Clinical Sciences Lund, Lund University, Skåne University Hospital, Lund, SE, 221 85, Sweden

**Keywords:** Overweight, Obesity, Preschool, Child, Parent, Intervention study

## Abstract

**Background:**

As the rate of overweight among children is rising there is a need for evidence-based research that will clarify what the best interventional strategies to normalize weight development are. The overall aim of the Lund Overweight and Obesity Preschool Study (LOOPS) is to evaluate if a family-based intervention, targeting parents of preschool children with overweight and obesity, has a long-term positive effect on weight development of the children. The hypothesis is that preschool children with overweight and obesity, whose parents participate in a one-year intervention, both at completion of the one-year intervention and at long term follow up (2-, 3- and 5-years) will have reduced their BMI-for-age z-score.

**Methods/Design:**

The study is a randomized controlled trial, including overweight (n=160) and obese (n=80) children 4-6-years-old. The intervention is targeting the parents, who get general information about nutrition and exercise recommendations through a website and are invited to participate in a group intervention with the purpose of supporting them to accomplish preferred lifestyle changes, both in the short and long term. To evaluate the effect of various supports, the parents are randomized to different interventions with the main focus of: 1) supporting the parents in limit setting by emphasizing the importance of positive interactions between parents and children and 2) influencing the patterns of daily activities to induce alterations of everyday life that will lead to healthier lifestyle. The primary outcome variable, child BMI-for-age z-score will be measured at referral, inclusion, after 6 months, at the end of intervention and at 2-, 3- and 5-years post intervention. Secondary outcome variables, measured at inclusion and at the end of intervention, are child activity pattern, eating habits and biochemical markers as well as parent BMI, exercise habits, perception of health, experience of parenthood and level of parental stress.

**Discussion:**

The LOOPS project will provide valuable information on how to build effective interventions to influence an unhealthy weight development to prevent the negative long-term effects of childhood obesity.

**Trial registration:**

ClinicalTrials.gov NCT00916318

## Background

It is a well-established fact that overweight and obesity is an increasing problem among children worldwide [1]. In 10-year old children born 1990 in the western parts of Sweden, overweight was twice as common, and obesity four times as common compared to among children born fifteen years earlier [2]. In 2008, 17% of 7-9-year-old Swedish schoolchildren in a nationally representative survey were overweight including 3% with obesity [3].

The arguments to prevent and treat childhood obesity are many. Overweight and obesity in childhood is a risk factor for overweight and obesity later in life, and the risk of persisting overweight is increasing the older a child gets [4-6]. There is a strong association between obesity and adult chronic disease [7,8] and individuals who are overweight or obese during childhood or late adolescence seems to have an increased mortality risk in adulthood compared to individuals with normal weight [9,10]. Moreover, obese children show reductions in global self-esteem and quality of life compared to children with normal weight [11].

The etiology of the rising incidence of overweight and obesity worldwide is not known although professionals have agreed that the origin most likely is multifactorial. There is a strong genetic influence; some studies indicate that more than 50% of variations in bodyweight can be explained by inheritance [12]. Still, the development of obesity requires several additional factors. Those can be described by using the Ecological systems theory (EST), which states that to understand the development of certain characteristics, one must consider the context, or ecological niche an individual is situated in. The ecological niche consists of a combination of the social environment, and the characteristics of the person itself. In the case of a young child, an important part of the ecological niche would be the family, but one would also have to take into account the preschool and the society where the child lives, as well as the characteristics of the child itself. To understand the development of childhood overweight according to the EST, one has to consider child characteristics (e.g. gender and genetic susceptibility to weight gain) and child risk factors (e.g. behavioral pattern such as preference for high energy density food, level of physical activity and sleep-pattern) as well as parental style practices (e.g. child feeding practices, parent monitoring child TV viewing, parent encouragement of child activity), family characteristics (e.g. peer and sibling interaction) and other parental factors (e.g. parental stress, and serious life events [13,14]. Thus, to understand the etiology and to evaluate the intervention effects it seems important to measure variables that have to do with child characteristics and risk factors as well as parenting characteristics and parenting styles.

Although the motive and the positive effect of intervening to prevent or treat childhood obesity seems clear, there is a lack of significant evidence to clarify to practitioners and policymakers what are the best interventional strategies [15]. Many of the interventions to date have focused on school-aged children and adolescents and often the interventions have been conducted in school settings. Several studies have had a short intervention time and many do not evaluate the long-term effects on studied parameters. For children under the age of five there is even less research on the effectiveness of different weight management schemes [15,16].

### Theoretical framework and structure of LOOPS- Lund overweight and obesity preschool study

When considering factors that could be important in influencing the outcome of obesity interventions, age seems to be relevant. Studies indicate that young age at intervention is a strong predictor for positive outcome, both in the short and long term [15,17-19]. This is in consistence with qualitative research, which shows that parents ask for early interventions that focus on the preschool years, a period when behaviors and habits are shaped [20]. It also seems that, to be effective, interventions should focus on long-term lifestyle changes in order to promote a more healthy energy balance [21]. In addition, interventions that are family based appear to have greater effect than interventions focusing merely on the obese child [21,22]. There is an overall positive effect of engaging the parents [23] and an even greater effect if the intervention induces changes in the whole family system instead of only in the child. When targeting the parents, the focus should be on supporting the parents in general parenting and family functioning instead of the traditional weight reduction strategies where the role of the parent is supervising the eating of the child without assisting them with strategies on how [21,24]. Interventions addressing exclusively the parents of an obese child have indicated positive outcomes, in some cases even more positive than those addressing both child and parents [25,26].

*LOOPS* is designed for preschool children, aged 4–6 years. The focus is on the parents of the overweight or obese child since we assume that children in this age group have very little influence over their eating and exercising habits, which do more reflect the habits of the whole family.

When designing the LOOPS intervention, we assumed that parents most often have a general knowledge about how to live healthy. In everyday life, however, there is a gap between how people intend to live and how they actually live their lives [19]. The problem is not a lack of knowledge about a healthy lifestyle, but that it is hard to accomplish lifestyle changes, and even harder to maintain a change over time. Since dealing with obesity will need lifelong attention to food habits and an active lifestyle, it is important to make early childhood interventions before the overweight becomes a major concern.

The intervention starts with a 2-hour lecture, *General facts about overweight in children (GFO),* performed by health professionals. The parents also get access to a website, *Healthy Children* (HC), with general information about diet and exercise recommendations. All information is in consistence with national guidelines and recommendations for preschool children. The purpose of the website and the lecture is to offer all parents the same basic knowledge about the importance of healthy food and physical activity.

Aside from the lecture and website, the parents are invited to attend group meetings with the general purpose of supporting the parents to accomplish preferred lifestyle changes, both in the short and long run. To evaluate the effect of various supports, the parents are randomized to either meetings led by a clinical psychologist or to meetings led by an occupational therapist. The two interventions have in common that they aim to support the parents to make long-term lifestyle changes that will lead to a healthier eating and a more active lifestyle. The difference is that they in part have different theoretical frameworks and ways in which they influence the parents to accomplish the changes. The interventions also differ in the number of participants in group activities and intensity in parental support.

The intervention arm led by a clinical psychologist, *Better balance everyday – parenthood and lifestyle (BBE),* aims to accomplish change by: 1) giving parents the possibility to reflect on their knowledge of healthy habits and on the discrepancy between how they want to live and how they actually live their lives, 2) in interaction with parents in similar situations, discuss possible solutions to some of the obstacles for living as healthy as one would like, and 3) to motivate parents to try new ways in their everyday life both concerning lifestyle and parenting. Since it has been shown that parental insecurity in relation to the parental role is more common than to worry about medical or physical issues in relation to child overweight, the intervention contains a parent support section, in addition to the techniques with special focus on food and physical activity.

The intervention arm led by an occupational therapist, *Lighter Living (LiLi)*, is based on the theory that alterations in the parents’–everyday life will induce changes that will gradually lead to a normalization of their children’s weight. Through the intervention, the participants learn to apply tools to identify possible changes in their patterns of daily activities that will lead to a healthier lifestyle in the family. For example, the parents analyze their own patterns of daily activities in order to identify needs and strategies for change. The process of accomplishing change is supported through experiences of daily activities performed at group sessions and in the parents’ own context. Thus, the individual’s reflection on their own doing is viewed as a prerequisite for sustainable changes in their everyday life.

### Objectives of LOOPS- Lund overweight and obesity preschool study

The overall aim of LOOPS is to evaluate if a family-based intervention, aiming at preschool children with overweight and obesity, has a long-term positive effect on the weight development of the targeted children. A secondary aim is to evaluate the different interventional strategies and to compare different sub groups to find if certain factors, such as child psychological and physiological characteristics and parental weight or SES are related to treatment outcome in the different interventions. The hypothesis is that preschool children with overweight and obesity, whose parents participate in the one-year intervention, both at completion of the one-year intervention and at long term follow up (2-, 3- and 5-years) will have reduced BMI for age z-scores compared to inclusion.

Specific objectives of the study are: 1) Find predictors that indicate treatment outcome. 2) Analyze the activity pattern and energy intake of preschool children with obesity and overweight and, in a subgroup of children, analyze if the activity pattern is changed by intervention. 3) Analyze the sleep duration of obese and overweight preschool children and, in a subgroup of children, analyze if the sleep duration is changed by the intervention. 4) Analyze insulin resistance, adipokines and inflammatory parameters as well as gut micro flora and to compare the results with those of normal weight children as well as to the results after one-year intervention. 5) Analyze ghrelin levels before and after a meal and in a subgroup of children analyze if the levels are changed by intervention. 6) Analyze how the intervention might affect the level of parental stress, the parents’ perception of the child and other motivational factors.

## Methods

### Study procedure

#### Study design

**Figure 1:**
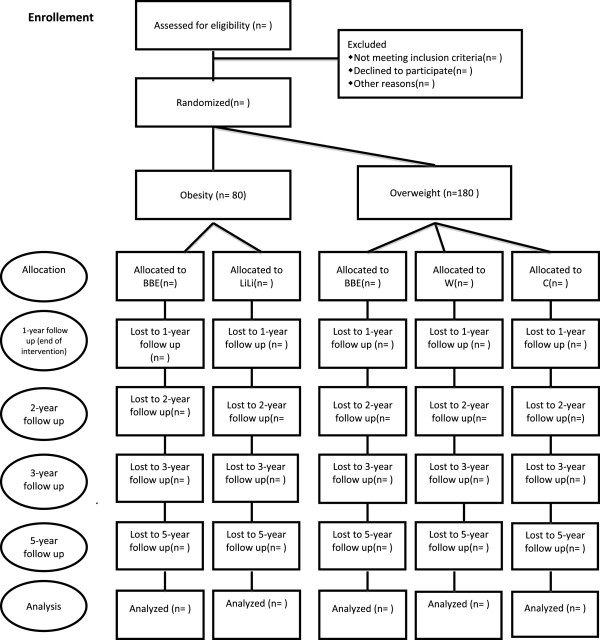
**Study design.** Showing the design of the trial. n=number of participants.

The study is a combination of a randomized controlled trial and a discontinuity regression design and is including 4–6 year old children with overweight and obesity. The children are randomly allocated to either one of the different intervention groups or a control group (Figure 1). Children with overweight are not randomized to the intervention strategy *LiLi* though it was considered unnecessary intense for this group. Children with obesity are not randomized to intervention with merely access to *Webbsite* since it is thought not to be intense enough. Children with obesity are not randomized to the control group since it was not found ethical when parents are seeking medical attention to address the problem. A wait-list control group is not considered an option since we plan to follow the long term BMI development of the children. The trial will be conducted and reported in agreement with the CONSORT recommendations [27]. The study was approved by the Regional Ethical Review Board in Lund, Sweden on May 8^th^ 2008 (Dnr 159/2008) and is registered at ClinicalTrials.gov (NCT00916318).

#### Setting and participants

LOOPS is set in Central Skåne Health district that consists of ten municipals in the south of Sweden with a total of 300 000 inhabitants. The largest municipal, Lund, is a University town of approximately 100 000 inhabitants whereas several of the other municipals are smaller with diverging socioeconomic profiles**.** The annual birth rate in Central Skåne Health district is around 3800 births. Due to the unique Swedish system where almost all children (95-99%) are monitored regularly at Child Health Monitoring Centres (CHMC), children with overweight and obesity can be identified at the routine visit at 4- or 5-years of age. The registered nurses at CHMC have been informed about LOOPS and to refer children with overweight and obesity to the study at the Children’s Hospital. The selection bias for children not referred to the intervention will be estimated and the number of patients referred, but not wanting to participate will be monitored. Exclusion criteria’s are if the parents don’t understand written and spoken Swedish well enough to participate in group activities.

#### Trial structure

**Figure 2:**
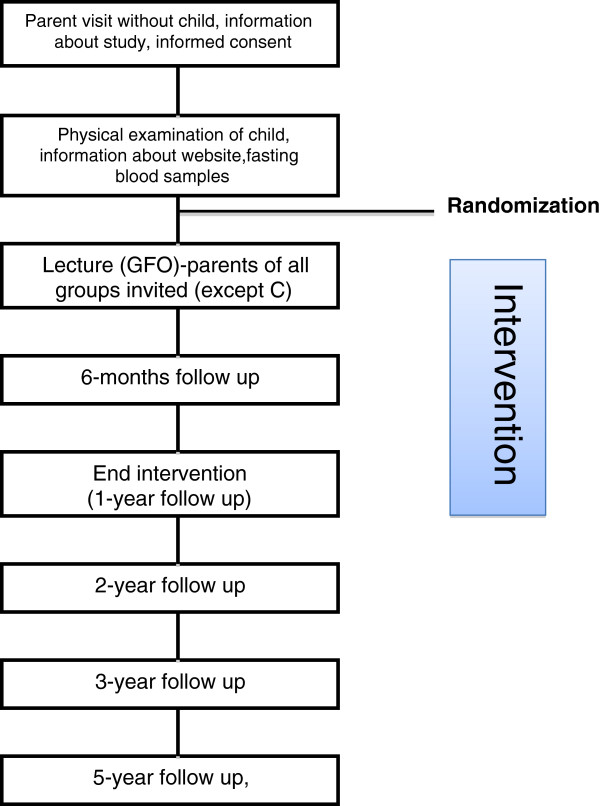
**Flow chart.** Showing the flow of participants through the trial and points of follow up in relation to the intervention.

If a child is referred to LOOPS, the parents get a letter with written information and are invited to come to the Children’s Hospital in Lund for a visit, the first time without their child (Figure 2). At this consultation the parents get more information about the trial and, if wanting to participate, sign the informed consent and answers a questionnaire about the child (heredity, development, previous health record, activity pattern, eating behaviour etc.). The parents also meet with a registered dietitian to get information about dietary registration and accelerometer measurements. Short after, the child comes to the centre with the parents for a clinical examination by a pediatrician, and to measure weight, length, waist circumference and blood pressure. Fasting blood samples (Table 1) are collected. The weights and lengths of the parents are measured. The child is randomized to one of following groups (Figure 1):

• *Children with overweight: 1)* Control group (C), *2)* Parents get access to website (HC), *3)* Parents get access to webpage and participate in ”Better balance everyday – parenthood and lifestyle” (BBE)

• *Children with obesity:* 1) Parents get access to webpage and participate in ”Better balance everyday – parenthood and lifestyle”” (BBE, 2) Parents get access to webpage and participate in “Lighter Living” (LiLi)

• All parents (except C) are invited to the lecture “General facts about overweight in children” (GFO).

**Table 1:** Showing data collected for all children/parents at different time points

**Measurements**	**Inclusion**	**6-months follow up**	**1-year follow up (end intervention)**	**2-year follow up**	**5-year follow up**
Weight/length/waist circumference	X	X	X	X	X
Blood pressure	X		X		
Parental weight/length	X		X		
fP-glucose, fS-insulin, fP-C-Peptid, fS-Proinsulin, B-HbA1c	X		X		
fS-ghrelin	X		X		
Ghrelin 1 hour postprandial	X		X		
P-glucose, S-insulin 1 hour postprandial					
X		X			
P-TSH, P-T4, P-T3	X		X		
P-ASAT, P-ALAT	X		X		
fP-kolesterol, fP-LDL-kolesterol, fP-HDL-kolesterol, fP-triglycerider	X		X		
Incretins, adipokines and cytokines	X		X		
Faecal samples (gut microbiota)	X	X	X		
F-calprotectin	X	X	X		
Accelerometer (SENSEWEAR) 3 days	X		X		
Dietary registration 4 days	X		X		
Questionnaire	X		X		

#### Intervention components

##### Lecture “general facts about overweight in children” GFO

The purpose is to give all parents the same basic knowledge about overweight and obesity in children. The lecture is 105 minutes (including a 15 minutes break) and is given in evening time to facilitate for both parents to attend. Each professional gives a short “lecture” about the topic and there is time to ask questions. The following elements are included: 1) A pediatrician is giving definitions of overweight in children, showing trends in society and is talking about long- and short time health consequences of being overweight or obese. 2) An authorized dietitian, specialized in pediatric nutrition, is giving the parents advice on healthy food for the age group, focusing on in between mealtime snacks and portion size. 3) An authorized clinical psychologist is talking about psychological perspectives on obesity; such as the importance of food and mealtimes, different meanings put into “limit-setting”, the importance of habits and the difficulties and obstacles in changing them. The value of the family context and finding ways to handle the problem in a way suitable to ones own family is discussed. 4) An authorized occupational therapist is presenting aspects of the everyday life and the daily activities of families. Parents are given the possibility to reflect on stress in dual working families with children, resulting in low physical activity and less time for cocking and mealtime.

Between and after the lectures there are discussions about how routines and habits develop and how these can be changed by time saving strategies.

##### Website “healthy children”

The purpose is to replace all information provided in paper form (e.g. brochures, hand-outs). The website is locally adapted and has been created especially for parents of children in the intervention program and is accessible only for them. It contains facts about overweight and obesity in children, information about healthy food, for example recommendations on portion size and recipes, and suggestions on outdoor activities to increase physical activity. It also contains a function where parents can write questions to a pediatrician, a dietitian, a psychologist or an occupational therapist. The answers are anonymous and published on the website. In addition, the webpage contains a forum where parents can interact with each other. All information on the website is based on national guidelines and recommendations for preschool children whether overweight or not.

##### ”Better balance everyday – parenthood and lifestyle, BBE”

Parents of 6**–**10 children are invited to each group. The group leader is an authorized clinical family psychologist. The groups meet for six two-hour sessions over 12 months. The focus of the sessions are: 1) ”Why don’t we live as healthy as we know we should? – Identification of obstacles and possibilities in everyday life”. 2) ”How can I get my child (and the rest of the family) to live more healthy? – Discussion about habits, models, rewards and the power of repeating/trying something a thousand-times”. 3) ”How do you say no to the wishes and demands of your child? – Children’s and parents’ needs”. 4) ”How do you keep a healthy lifestyle? – Discussion about goal setting and motivation”. 5) Tasty and healthy food – a practical and inspiring cooking event”. The sessions follow a structure including: 1) a discussion about experiences of changes since the last session, 2) the introduction of the theme for the day with a short presentation by the group-leader, 3) discussion of the theme sometimes based on vignettes of common situations and 4) summery and new “home-work” assignment.

The parents are also, in two occasions, invited to attend a 3-hour cooking class where they practice to cook healthy food in cooperation with a home economics teacher.

##### “Lighter living, LiLi”

Parents of 3–5 children are invited to each group. The group leader is an authorized occupational therapist and every session is manual directed. The groups meet for thirteen two-hour sessions over 12 months. The first sessions involve activity self-analysis and identification of resources and obstacles in the families’ everyday life. Both parents are encouraged to write 24-hour Time-Use diaries in the first week of the program and at seven additional occasions during the program. The diaries are, with the help of a computer program, transformed to graphs showing the distribution of activities during the day, for example what was done, where the individual were, with whom and food intake during the day for documentation. These graphs then serve as starting points for reflection among the parents. In the following sessions the participating parents implement strategies for changing the everyday life that they themselves formulated in order to achieve their own goals for change in benefit for their children. In the group sessions they have the opportunity to evaluate their alterations in everyday life and reflect upon their experiences. The last two sessions aim at refilling and following up the themes from previous sessions. The LiLi program is planned as a course and the basic fundaments in the program are client education, activity self-analysis, experiences of participation in activity in group sessions and homework tasks (e.g. new routines together with their children). The education part implies shorter seminars regarding different aspects of daily activities, its structure and values and challenges for changes. The activity self-analysis is focusing the content and organization of a pattern of daily activities and is intended to result in a motivation for parents to change and to set up goals and strategies for accomplishing these changes.

##### Control group

The parents and children are monitored and followed according to the flow chart (Figure 2), except not participating in the interventional parts. They are monitored with the same measurements as all other groups (Table 1).

#### Randomization

The randomization is blocked in series of ten (obese) or fifteen (overweight) to ensure all groups a steady flow of participants. The randomization is stratified so that children who have at least one parent with obesity are randomized separately from children with both parents being normal weight or overweight. The allocation series is concealed in numbered envelopes.

### Measurements

#### Anthropometry

Weight, length and waist circumference is measured in a standardized way at inclusion, after 6 months, at the end of intervention and at 2-year follow up. The measurements are made by one of two registered pediatric nurses in the project. At 3- and 5- year follow up, a registered school nurse makes the measurements. BMI is calculated as weight (kg) divided by height (m) squared, BMI=kg/m^2^. We use the BMI Standard Deviation Scores (BMI-SD) to obtain age- and gender specific reference values for BMI [28]. Overweight and obesity is defined by iso-BMI according to the definition by Cole et al. [29].

#### Registration of physical activity

Physical activity, energy expenditure and sleep duration is measured using the SenseWear Armband version 3 (Body media, inc.). The armband contains a 3-axis accelerometer, a galvanic skin response sensor, a skin temperature thermometer and a device to measure heat flux from the body. The armband is placed on the upper right arm and is worn for four consecutive days at inclusion and after the one-year intervention. The parents receive oral and written information about how to use the armband.

#### Registration of nutrition

A four-day dietary registration is made at inclusion and after the one-year intervention. Before the first registration, the parents meet with an authorized dietitian to get oral and written instructions on how to perform the registration. The time of day for every meal is reported and the amounts of ingested foods and beverages are estimated and registered in absolute amounts in a diary. The result is analyzed using the computer program Dietist XP.

#### Parent questionnaire

Both parents are asked to fill out a questionnaire at inclusion and after the one-year intervention. The questionnaire contains the following parts: 1) Questions on food and exercise habits, socioeconomic factors, parental practices and parental health. 2) Assessment of hassles and uplifts in everyday life, THU-3 and the Mastery scale [30,31]. 3) The Strength and Difficulties Questionnaire (SDQ) that asks questions about children’s psychological health [32]. 4) The Swedish Parenthood Stress Questionnaire (*SPSQ*). The materials contained in SPSQ are partly adapted and modeled after the Parenting stress Index [33,34].

#### Website

When enrolled in the study, each parent is provided with a unique username and password. The log-on rate is registered as well as the number of pages downloaded by each user. Comments on the forum wall are registered as well as questions to different experts.

### Outcomes

#### Primary outcomes

The primary outcome variable is change in BMI z-score of the child, which is measured at referral date, inclusion, after six months intervention and at the end of intervention. Follow up will be performed one, two and four years after end of intervention.

#### Secondary outcomes

The secondary outcome variables are change in children dietary and exercise patterns as well as change in waist circumference, insulin resistance, dietary hormones and fecal micro flora (Table 1). Other secondary outcomes are parent change in BMI and parent perception of their own health, parent stress as well as child feeding and exercise habits.

### Statistical analysis

The design of the study is a combination of a randomized controlled study and a discontinuity regression design. A relevant difference in BMI of the children before and after intervention is set to 0.8 SD. With a power of 80–85% 40 – 45 individuals who fulfill the treatment are needed in each group if comparisons in the same intervention group. Every child is its own control, in the matter of comparison between the BMI curve of the child before and after the intervention.

For comparisons between two groups 25–30 individuals are needed per group. With 25 children in each group, there will be 80% chance (β=0.8) with a two-sided significance level 0.05 (α=0.05) to detect a difference of 0.8 SD between the groups. With a group size of 60 children per intervention group for the overweight children (three groups) a loss of 20% can be tolerated. With a group size of 40 per group for the obese children (two intervention groups) a loss of 25% can be tolerated.

## Discussion

In this paper, we describe the design of family-based intervention program targeting parents of overweight and obese preschool children. The aim of the intervention is to stop further unhealthy weight gain in participating children to prevent the long-term negative effects of obesity. If the intervention is found successful, the protocol could be implemented at a broader scale in the Child Health Monitoring Centers. The intervention has several strengths and will provide valuable information about family interventions for obesity in preschool children.

First, the intervention is built on a theoretical model that emphasizes the importance of influencing the whole family to accomplish a change. Although the primary outcome variable is change in child BMI z-score, several registered variables will detect a change in the parents’ behavior and in that way show further effects of the intervention. For example, parents BMI is measured before and after intervention and parents answer questions about their exercise habits, intake of fruits and vegetables and health, as well as questions about their experience of parenthood, both strengths and weaknesses.

Second, the intervention does not focus on the classical weight management strategies such as strictly reducing calorie intake and increasing exercise for the obese individual, but is more addressing long-term changes of family habits such as family activity pattern and family eating habits. Recommendations on food and exercise are based on general evidence-based national guidelines and are applicable to all members of the family whether obese or not. The information is made available to the parents who then find strategies to influence their child. The obese child does not, except from the necessary physical examination and occasional weight and length controls, participate in any part of the intervention. This strategy has the advantage of shifting focus from the obese child, reducing the risk of stigmatization. The focus is not in reducing the weight of the child but to accomplish long-term reductions in the velocity of weight gain that in the long run will normalize the BMI.

Third, the intervention is focusing on long-term changes and the effects will be monitored over time, at least four years post interventional which will contribute to the knowledge on how to build effective interventions that has a long-term effect.

Fourth, the intervention has a potential strength in being multidisciplinary, involving a multidisciplinary team of pediatricians, nurses, child psychologist, occupational therapist and nutritionist. The evaluation of the intervention will include a valuable comparison between the different interventional strategies. It will also include sub-group analyzes to find in certain interventional strategies are preferable to a particular sub-group of parents.

The intervention also has some weaknesses, both concerning the evaluation and the design of the study. One limitation is the initial recruitment of participants to the project where certain groups might be underrepresented. It is plausible to expect that parents of lower SES and educational level might have difficulties understanding the research project, thereby rejecting participation. This problem is partly avoided by the recruitment of participants through the Child Health Monitoring Centers where the nurses often have worked with the same family over several years and have built trust and confidence. Because of the practical difficulties with arranging interpretation of group sessions, children of parents not fluent enough in Swedish will be excluded from the project.

Another potential weakness is that measures of family habits and family functioning are based on reports from the parents themselves and not on objective evaluation from the researchers. Besides, the number of questions that are included in the questionnaire is limited by the parent’s willingness to participate if too time consuming.

Ethical consideration has to take into account not only the impact on the obese child itself but also the impact on the whole family since the intervention focus not only on the child. Since many overweight children become obese adults and obesity has a strong association to chronic disease, we state that treatment of the child is motivated. By addressing the whole family as described above, negative impact of the child is reduced. To motivate whole-family involvement, the intervention has to be based on general knowledge in healthy food and activities that all family members will benefit from, whether obese or not.

In conclusion, it is our belief that this study will provide valuable information on future intervention strategies and that it if successful, because of its close connection to the Child Health Monitoring Centers, can be easily implemented.

## Competing interests

The authors declare that they have no competing interests.

## Authors’ contributions

KTJ, MB and L-KE designed the study at large but all other co-authors were also engaged in designing different parts of the study. JÖ drafted the manuscript. All other co-authors provided comments on, and accepted the last version of the draft manuscript. All authors read and approved the final manuscript.

## Pre-publication history

The pre-publication history for this paper can be accessed here:

http://www.biomedcentral.com/1471-2458/12/879/prepub
